# Use of 5-Fluorouracil-Soaked Bioamniotic Membranes in Trabeculectomy for Primary Open-Angle Glaucoma: A Retrospective Analysis

**DOI:** 10.1155/2017/2698975

**Published:** 2017-08-06

**Authors:** Chenming Zhang, Min Wu, Jianrong Wang, Miaomiao Zhang, Xu Wang, Wei Liu

**Affiliations:** Eye Research Institute, Jinan Eye Hospital, No. 148 Jingyi Road, Jinan, Shandong 250001, China

## Abstract

The study is a retrospective analysis of 51 patients (76 eyes) with primary open-angle glaucoma (POAG) who admitted to our hospital from 2008 to 2010 to analyze the efficacy of trabeculectomy in combination with 5-fluorouracil- (5-FU-) soaked amniotic membranes for the treatment of POAG patients. Among them, 30 patients (41 eyes) were treated with trabeculectomy in combination with 5-FU-soaked amniotic membrane and 21 patients (35 eyes) were treated with trabeculectomy in combination with MMC. Preoperative and postoperative intraocular pressures (IOP), cup/disc ratio, visual acuity and postoperative macular OCT, complications, treatment, and number of corneal endothelial cells were measured, recorded, and analyzed. At the end of 2 years of follow-up, IOP of 36 (87.8%) eyes of patients in the 5-FU group and IOP of 28 (80%) eyes of patients in the MMC group were ≤21 mmHg and patients in the 5-FU group had more stable IOP than patients in the MMC group. During the two years of follow-up, the visual acuity of 22 (53.7%) eyes in the 5-FU group remained unchanged or even improved. Trabeculectomy in combination with 5-FU-soaked biological amniotic membranes can be a surgical option for POAG patients.

## 1. Introduction

Trabeculectomy is the main surgical treatment of glaucoma. Channel blockage due to postoperative scarring of the filtering bleb is the major cause of surgical failure. Adaption of implants such as amniotic membrane and biological glue and the use of antiproliferative drugs such as antiproliferative drugs 5-fluorouracil (5-FU) and mitomycin C (MMC) in the operation have significantly reduced the failure rate [1]. The special microstructure of the amniotic membrane renders it as a “natural sustained drug release system” [2]. Therefore, we used 5-FU-soaked amniotic membrane in trabeculectomy and investigated the formation of filtering channels after surgery using ultrasound biomicroscopy (UBM) and evaluated its clinical efficacy for primary open-angle glaucoma (POAG). Here, we report the clinical outcomes of 5-FU-soaked amniotic membrane in trabeculectomy for POAG patients from an eye specialty hospital in Jinan, China.

## 2. Materials and Methods

### 2.1. Patients and Treatments

A total of 76 eyes from 51 POGA patients admitted to our hospital from 2008 to 2010 were retrospectively analyzed. Among them, 30 patients (41 eyes) were treated with trabeculectomy in combination with 5-FU-soaked amniotic membrane and 21 patients (35 eyes) were treated with trabeculectomy in combination of MMC treatment. All operations were performed by the same surgeon (GBL). Patients who had surgery before were excluded. The protocol was reviewed and approved by the Institutional Ethics Committee of The Second People's Hospital of Jinan. Each participant provided written informed consent before any study-related examination or procedure was performed, and the study adhered to the tenets of the Declaration of Helsinki. Patients were randomly assigned to receive either trabeculectomy in combination with 5-FU-soaked amniotic membrane or trabeculectomy in combination of MMC treatment according to a computer-generated randomization list.

Patients' age, family history, pre- and postoperative (at days 1 and 3, week 1, and months 1, 6, 12, and 24) IOP, cup/disc ratio, number of corneal endothelium cells, and visual acuity, as well as postoperative complications, were collected and evaluated using corresponding technologies such as fundus optical coherence tomography (OCT), ultrasound biomicroscopy (UBM), slit-lamp examination, and follicular morphology.

Standard 5-FU and 5-FU injection solutions (25 mg/mL) were from Xudong Haipu Pharmaceutical Co. Ltd (Shanghai, China). HPLC grade methanol and ultra-pure water were from J.T. Baker Company (USA). Lyophilized and Co^60^ sterilized bioamniotic membranes with a size of 1.5 × 1.5 cm and free of hepatitis B, hepatitis C, syphilis, HIV, and other pathogens were from Ruijin Medical Devices Co. Ltd (Jiangxi, China). The amniotic membranes were soaked in 30% glycerol. After prefrozen for 10 h at 0°C to −56°C, the membranes were lyophilized at −10°C to −56°C till their water content reached 1 ~ 2%, repacked, sterilized by gamma irradiation from Co^60^. Before use, the membranes were soaked in 5-FU solution (25 mg/mL) for 3 min and then rinsed with normal saline solution for 2 s [2].

### 2.2. Surgical Methods

A conjunctival flap was made on the base of fornix on the upper margin of the temporal quadrant. After hemostasis, a scleral flap of 4 × 6 mm with ½ thickness of sclera was put from the corneal margin into the transparent cornea region and a trabecular tissue (2 × 3 mm) and a portion of peripheral iris below the scleral flap were resected. For patients in the 5-FU group, the prepared amniotic membrane was implanted into the scleral flap with skin facing up, then the sclera and the bulbar conjunctiva were closed in turn. For patients in the MMC group, a cotton sheet of 3 × 4 mm, which had been soaked in 0.4 g/L MMC solution, was placed under and above the scleral flap. After 3 min, the cotton sheet was removed, and the scleral flap and its surrounding tissues were rinsed immediately with 200 mL normal saline solution. The flaps and conjunctival incisions were closed using 10/0 nylon and 8/0 vicryl sutures, respectively. Then, 0.1 mL hyaluronic sodium solution (hyaluronan, HA) was injected into the anterior chamber through the lateral incision, and tobramycin and dexamethasone eye ointments were applied to the conjunctival sac. The eye was covered and treated with antibiotic eye drops (0.3% tobramycin eye drops, 4 times per day; tobramycin dexamethasone eye ointment, once per night).

### 2.3. Efficacy Evaluation

#### 2.3.1. Definition of Success

Complete success was defined as IOP ≤ 21 mmHg at 2 years of postoperation without use of any IOP-lowering drug or other measures such as subconjunctival injection of 5-FU or follicular needle revision. Partial success was defined as IOP ≤ 21 mmHg at 2 years of postoperation with the use of IOP-lowering drugs or other measures. Failure was defined as IOP > 21 mmHg with the use of IOP-lowering drugs or other measures.

#### 2.3.2. Vision Assessment

Visual acuity was examined using the standard logarithmic visual acuity chart. Visual acuity improvement was defined as promotion for 2 or more lines. Visual acuity decline was defined as decrease for 2 or more lines no change or not to change less than 2 Walker change. Unchanged visual acuity was defined as promotion or decline for less than 2 lines. In addition, for patients with visual acuity less than 0.1, an increase or decline of 0.02 in the standard chart was defined as improvement or decline. The best postoperative corrected visual acuity was used for comparison.

#### 2.3.3. Filtering Bleb Morphology

Filtering blebs were classified as previously reported [3]. Type I is a thin cystic type; type II is a thick diffuse type; type III is an absent type; and type IV is an encapsulated type. Among them, types I and II are functional and types III and IV are nonfunctional.

### 2.4. Statistical Analysis

Continuous variables were analyzed using the unpaired Student's *t*-test. The categorical variables were analyzed using the chi-square test. Discrete variables were analyzed using Fisher's exact test. A *p* < 0.05 was considered significantly different. All analyses were preformed using SPSS software (version 16.0, SPSS Inc., Chicago, IL, USA).

## 3. Results

### 3.1. Preoperative Baseline Characteristics

As shown in Table 1, the baseline characteristics were similar in both groups. There were no statistically significant differences in the mean preoperative IOP, age, and gender between patients in 5-FU group and patients in MMC group.

### 3.2. Postoperative IOP and Success Rate

**Figure 1:**
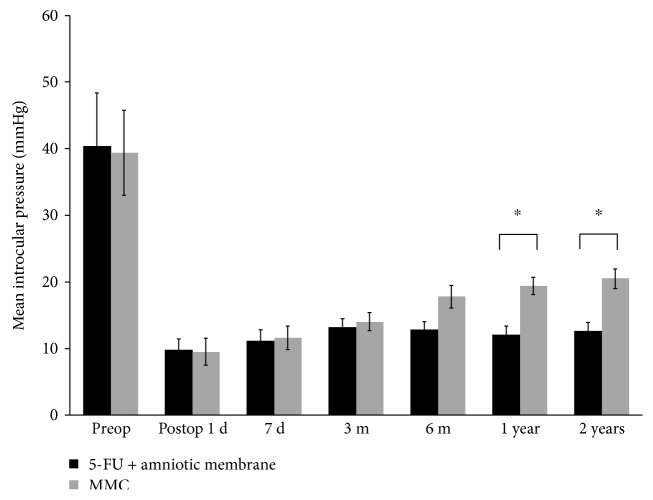
Intraocular pressure in patients treated with trabeculectomy in combination with either 5-FU-soaked amniotic membrane or MMC. ^∗^The intraocular pressures at the first and second year of follow-up were significantly different between the MMC group and the 5-FU group (*p* = 0.000).

Among the 41 eyes of patients in 5-FU group, IOP of 36 (87.8%) eyes at 2 years of postoperation was ≤21 mmHg and that of 5 (12.2%) eyes was >21 mmHg without taking any antiglaucoma medication. Among the latter, the IOP of 3 (7.3%) eyes was reduced to 21 mmHg after treated with IOP-lowering drugs while the IOP of 2 (4.9%) eyes was still >21 mmHg after treatment. These 2 eyes were subjected to reoperation. By comparison, among the 25 eyes of patients in MMC group, the IOP of 28 (80%) eyes was ≤21 mmHg and that of 7 (20%) eyes was >21 mmHg without taking any medicine. Among the latter, 3 (8.6%) eyes after drug treatment had IOP reduced to 21 mmHg and 4 (11.4%) eyes still had IOP > 21 mmHg and were subjected to the 2nd surgery. The average IOP of patients in the 5-FU group at 2 years of postoperation was significantly lower than that of patients in the MMC group (*p* < 0.05), indicating that 5-FU-soaked amniotic membrane is more effective in reducing IOP than MMC and during the 2 years of follow-up; IOP of patients in the 5-FU group was more stable than that of patients in the MMC group (Figure 1).

The complete success rate and partial successful rate were 87.8% and 12.2%, respectively, for patients in the 5-FU group and 80% and 20% for patients in the MMC group, showing no significant difference.

### 3.3. Filtering Bleb Morphology

**Figure 2:**
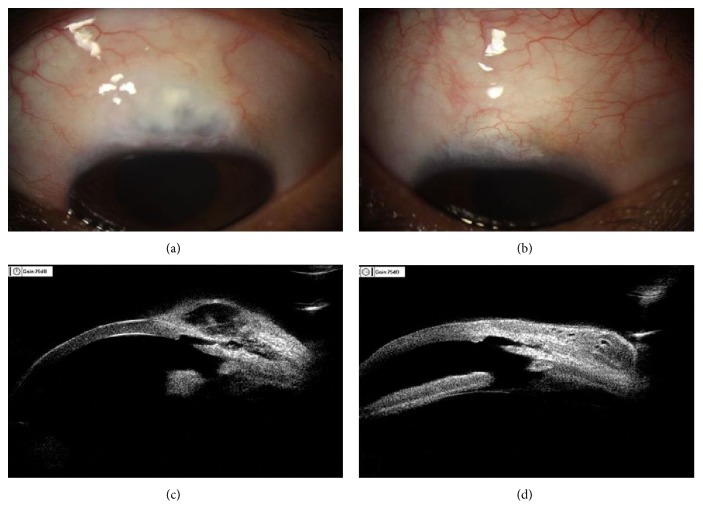
Slit lamp and UBM images of eyes in POAG patients treated with trabeculectomy combined with 5-FU-soaked amniotic membrane (a, c) and MMC (b, d) at two years of postoperation.

The slit lamp imaging at 2 years of postoperation showed that most blebs of patients in the 5-FU group were uplifted, flattened, and diffused, while only partial blebs of patients in the MMC group were uplifted and encapsulated with a clear boundary (Table 2). Although the morphology of blebs of patients in both groups was functional, UBM imaging showed that the blebs of patients in the 5-FU group had clear filtering channels with obvious lacuna and proper thickness of bleb wall, suggesting the blebs were functional, while the blebs of patients in the MMC group had less clear channels with disordered echo and thickened wall thickness, indicating they were nonfunctional (Figure 2). Therefore, 5-FU-soaked amniotic membranes were better than MMC in forming functional filtering blebs. As shown in Table 2, at 2 years of postoperation, 7 (17.1%) eyes of patients in the 5-FU group had type III/IV blebs, while 8 (22.9%) eyes of patients in the MMC group had type III/IV blebs, showing significant difference.

### 3.4. Corneal Endothelial Cells

The number of corneal endothelial cells before operation was 1968 ± 246.3 cells/mm^2^ for patients in the 5-FU group and 1923 ± 238.2cells/mm^2^ for patients in the MMC group, showing no significant difference (*p* = 0.423). By contrast, the number of corneal endothelial cells at 2 years of postoperation was 1584 ± 210.4 cells/mm^2^ for patients in the MMC group, significantly less than that of 1747 ± 198.5 cells/mm^2^ for patients in the 5-FU group (*p* = 0.001), indicating that the use of 5-FU-soaked amniotic membrane results in less loss of corneal endothelial cells than the use of MMC.

### 3.5. Complications

Among the 41 eyes of patients in the 5-FU group, 2 eyes had early anterior chamber hemorrhage at day 1 of postoperation as presented as the anterior chamber height of <2 mm and the blood was absorbed in 3–7 days without giving any treatment; and one patient had shallow anterior chamber accompanied with choroidal detachment at day 1 of postoperation and recovered in 5 days after giving corticosteroid eye drops. Among the 25 eyes of patients in the MMC group, 3 eyes had shallow anterior chamber, 3 eyes had choroidal detachment, and 1 eye had filtering bleb leakage. The patient with filtering bleb leakage was treated with repair surgery, and follow-up at 3 months of postoperation with OCT showed no macular edema at fundus.

## 4. Discussion

Minimally invasive glaucoma surgeries (MiGs) [4] can reduce IOP. However, some of these procedures can only produce limited IOP reduction compared to trabeculectomy, the classical option for POAG patients. The main cause for the failure of trabeculectomy is the scar formation in the filtering channels [5]. The way to create an effective filtering channel is of paramount importance to improve the success rate of glaucoma surgery. Various antidrainage device and antiproliferative drugs have been widely used in surgery to improve the success rate [6]. However, use of antiproliferative drugs also results in some complications, such as conjunctiva leak and secondary infection [7]. To avoid these complications and improve operative efficacy, implants such as amniotic membrane are being used [8, 9]. Studies also showed that combined use of antiproliferative drugs and implants might lead to higher success rate and reduced complications in bleb surgery [10, 11]. 5-FU is a fluorouracil derivative where the uracil at position 5 is substituted by hydrogen and could affect DNA synthesis in all cell cycles. Study [12] showed that in cultured cells, 5-FU could inhibit fibroblast proliferation, especially in the inflammatory stimulation period. When exposed to high concentration of 5-FU, the proliferation of fibroblasts in the target tissues is strongly inhibited. Currently, 5-FU is used as an adjuvant for bleb surgery in patients with glaucoma to improve the success of filtering surgery. Currently, the drug is mainly given by frequent subconjunctival injection after surgery, resulting in too high peak dose and possible toxicity to cornea and conjunctiva [13]. In addition, it is difficult to directly address the filtering channel, which to some extent limits its clinical application.

Amniotic membrane has a layer of collagen and can reduce the wound scarring and remove inflammatory cells, resulting in reduced inflammatory reaction and vascularization [14]. In addition, amniotic membrane can form a thin layer of pad graft in the sclera to prevent sticky scarring in filtering channels as a mechanical barrier, thus reducing the postoperative recurrence [15]. Therefore, amniotic membrane has been used in trabeculectomy. Ultrastructural studies of amniotic membrane show that the basement of the membrane is rich in collagen fibers and reticular fibers, which are interleaved into a mesh with intramesh gap of about 0.5–15 *μ*m [16]. The structure can accommodate a large amount of drugs and adsorb drug liquid as a drug reservoir. Bioamniotic membrane is self-degradable to gradually release drugs over a long period of time that maintains effective local drug concentration, and it is therefore an excellent partially controlled drug delivery system. Any particles with a size less than the micropore in the membrane can be stored in it. Studies have shown that many drugs can travel through the bioamniotic membrane. The size of 5-FU particles is about 180–280 nm, which is far less than that of the amniotic membrane pore. Therefore, once the membrane is soaked in 25 g/L 5-FU solution, it will contain certain amount of the drug molecules. After trabeculectomy, the membrane underneath the scleral flap is gradually dissolved and absorbed. 5-FU stored in the membrane is gradually diluted and released, which inhibits the proliferation of cells in filtering channels together with the bioamniotic membrane. Therefore, the bioamniotic membrane used in the study functions as a natural slow drug release device. Earlier study showed that the dissolution time of amniotic membrane is about 2 weeks after surgery, and the period to form the fibrous scar in the filtration area is 1 to 2 weeks after trabeculectomy [17]. Studies also found that 3–5 d after surgery are the peak period of fibroblast proliferation in the wound limbus of the sclera wound limbus and under the conjunctiva [18, 19]. Therefore, the use of 5-FU-soaked amniotic membrane in trabeculectomy can help maintain certain levels of 5-FU in the scleral flap and conjunctiva at an early stage of postoperation, which is obviously better than one-time application of the drug. In a 2-year follow-up study [19], the use of AMT with trabeculectomy was found to be safe and effective with an IOP-lowering effect and a reduced rate of postoperative complication comparable to that achieved with the use of MMC. Furthermore, AMT was proposed to have potential as an alternative to MMC in trabeculectomy.

It is well known that 5-FU is toxic to intraocular tissues. In this study, we evaluated its toxicity by investigating the changes in the number of corneal endothelial cells and retina macular area and found that at 2 years of postoperation, the number of corneal endothelial cells of patients in MMC group was significantly less than that of patients in 5-FU group, indicating that 5-FU-soaked amniotic membrane results in less loss of endothelial cells than MMC. In addition, application of 5-FU did not lead to macular cystic degeneration. In the study, 0.1 mL HA solution was injected into the anterior chamber through the lateral incision, which not only reduced the postoperative inflammation of the blebs, but also decreased the siphon effect of anterior chamber and 5-FU concentration in the anterior chamber.

In the previous studies, Beck et al. [20] applied MMC in trabeculectomy and reported that 22% patients had choroidal detachment, 8% had endophthalmitis, and 3% had hemorrhagic choroidal detachment, retinal detachment, and vitreous hemorrhage. In the Tube Versus Trabeculectomy (TVT) study [21], early postoperative complications occurred in 22 (21%) patients in the tube group and 39 (37%) patients in the trabeculectomy group. Compared with the success rate in the previous studies (e.g., 87% for implantation [22]), the operation success rate (87.8%, 36 eyes out of 41 eyes, as defined by controlling IOP < 21 mmHg) of joint use of trabeculectomy with 5-FU-soaked amniotic membrane at 2 years of follow-up in our study on 30 patients is satisfactory.

## 5. Conclusions

Use of 5-FU-soaked bioamniotic membrane is shown to maintain the patency of the filtration channel for a long time and reduce the postoperative IOP as well as related complications. Therefore, we believe that 25 mg/ml 5 FU-soaked amniotic membrane can increase success rate of trabeculectomy and is a safe and reliable surgical procedure for glaucoma and worth to encourage. Of course, the long-term complications associated with this surgical approach need to be further studied. Overall, application of improved bioamniotic membrane with antimetabolic drugs using bioengineering technology to inhibit fibrosis would be a future direction for glaucoma prevention and treatment.

## Figures and Tables

**Table 1:** Comparison of baseline data between patients in the 5-FU group and patients in the MMC group.

Parameter	5-FU group	MMC group	*P*
	*n* = 30	*n* = 21	
Mean preoperative IOP	40.39 ± 7.97	39.38 ± 6.35	0.461^a^
Age			
Mean	46.61 ± 10.05	48.43 ± 11.72	0.389^a^
Range	22–62	24–69	
Gender			
Male/female	19/11	11/10	0.421^b^

^a^Independent Student's *t*-test. ^b^Fisher's exact test.

**Table 2:** Morphological classification of filtering blebs in both groups.

Time postoperation	5-FU + amniotic membrane (*n* = 41)				MMC (*n* = 35)			
	I	II	III	IV	I	II	III	IV
1 y	0	37	3	1	0	29	4	2
2 y	0	35	5	2	0	27	2	6
